# Formation of Graphene Agglomerates in MOC and Their Negative Influence on Material Properties

**DOI:** 10.1021/acsomega.6c03968

**Published:** 2026-06-18

**Authors:** Anna-Marie Lauermannová, Adéla Jiříčková, Michal Lojka, Martina Záleská, Milena Pavlíková, Adam Pivák, Zbyšek Pavlík, Daniel Kytýř, Petr Koudelka, Petr Miarka, Ondřej Jankovský

**Affiliations:** † Department of Inorganic Chemistry, Faculty of Chemical Technology, 52735 University of Chemistry and Technology Prague, Technická 5, 166 28 Praha 6, Czechia; ‡ Department of Materials Engineering and Chemistry, Faculty of Civil Engineering, Czech Technical University in Prague, Thákurova 7, 166 29 Prague, Czechia; § Institute of Theoretical and Applied Mechanics, Czech Academy of Sciences, Prosecká 809/76, 190 00 Prague, Czechia; ∥ Institute of Physics of Materials, Czech Academy of Sciences, Žižkova 513/22, 616 00 Brno, Czechia

## Abstract

Magnesium oxychloride cement (MOC) is a promising low-carbon alternative to Portland cement; however, its application is limited by poor water resistance and insufficient understanding of microstructure–property relationships. This study presents a multiscale investigation of graphene (G) incorporation in MOC-based composites, explicitly linking microstructural features to mechanical and fracture behavior. Composites containing 0.05–1.0 wt % of G were prepared and compared with a graphene-free reference. Microstructure was characterized using XRD, OM, SEM–EDS, mercury intrusion porosimetry (MIP), and XCT, enabling quantitative assessment of porosity and graphene agglomeration. Mechanical performance was evaluated by compressive and flexural strength tests and crack mouth opening displacement (CMOD)-controlled fracture experiments. Hygric behavior was assessed through water absorption and residual strength. At low dosage (0.05 wt %), graphene improved compressive strength and reduced 24 h water absorption from 3.01% to 2.75%. In contrast, higher graphene contents (≥0.5 wt %) led to agglomerate formation exceeding 300 μm in size, increasing total open porosity from 9.3% to 11.5% and effective transport porosity from 5.1% to 12.4%. Despite this porosity coarsening, residual compressive strength increased up to 51.0 MPa, and the softening coefficient improved from 65% to 89%, indicating partial suppression of water-induced degradation due to graphene hydrophobicity. Fracture energy remained stable at ∼290 N·mm^–1^, confirming preservation of quasi-brittle behavior. The novelty lies in the integrated multiscale approach (XRD, SEM–EDS, MIP, XCT, CMOD), enabling quantitative differentiation of graphene agglomerates from air voids and direct structure–performance correlation. The results establish graphene dosage limits in MOC and demonstrate that performance is controlled by microstructural stability rather than graphene content alone.

## Graphical abstract

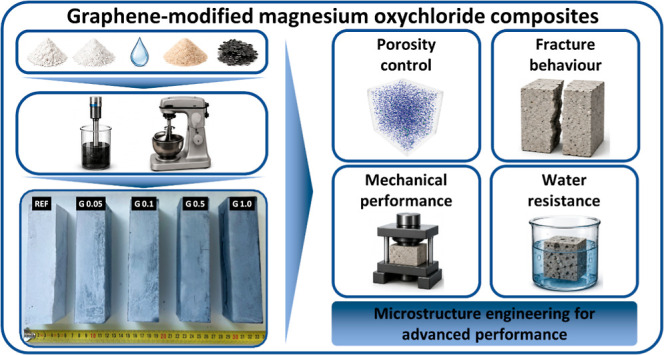

## 1 Introduction

The study of the utilization of carbon nanomaterials (CNMs) as functional additives in composites has emerged as a new field in the research of construction materials. This is primarily due to the excellent mechanical and thermal properties of these nanoadditives, which directly translate into the performance of the resulting composite. From this group of nanoadditives, especially 1D (carbon nanotubes and their derivatives) and 2D (graphene and its derivatives), CNMs are among the most researched options for the improvement of construction composites. These materials alone manifest very high values of tensile strength, Young’s modulus, and failure strain.^1,2^ Furthermore, these materials are characterized by their excellent thermal conductivity. Carbon nanotubes also exhibit increased thermal stability, making them suitable for high-temperature applications.^1,3–6^

The efficiency of material-property enhancement can be assessed from multiple perspectives. Considering the mechanical performance of the CNM-doped composites, it can generally be said that the addition of low percentages of CNMs can cause a significant improvement in mechanical parameters, such as compressive and tensile strength.^7^ From the previous studies, it can be summarized that the impact on mechanical performance is somewhat dimension-specific. Studies focusing on the utilization of 1D CNMs generally present significant improvements in tensile strength and dynamic modulus, whereas studies covering the utilization of 2D CNMs show their undeniable potential in enhancing the compressive strength of the resulting composite.^8–12^ Concerning the thermal properties, all types of CNMs help improve thermal conductivity and stability.^13–15^ Despite promising results, there are still some setbacks, which hinder the scalability of the CNM application in general practice. They can be summarized as the following three factors: (i) CNM dispersion quality, (ii) environmental impact, and (iii) cost efficiency. The difficulties in CNM dispersion stem from their hydrophobicity, which is influenced by their structural and chemical characteristics.^16–19^ This issue can be resolved by using specialized dispersion techniques or by the utilization of surfactants and other chemical agents.^20–22^ The environmental impacts relate to the environmental efficiency of CNM and CNM-doped composites production and also to the health risks connected with CNMs. It has been demonstrated that the overall environmental impact of CNM production can be offset by the composite’s prolonged durability.^23–27^ The health risks connected with CNMs can be offset by the utilization of a stable matrix, which prevents leakage of these materials into the environment.^28,29^ The cost-related difficulties were assessed in multiple reviews, showing the main obstacle to the scalability of this approach. However, recent studies suggest that as CNM research and development advances, costs decrease rapidly. Methods such as HiPco or laser scribing are cost-effective alternatives to conventional CNM production methods (arc discharge or chemical vapor deposition).^30–32^ The main factors influencing the cost are the used raw materials, the synthetic procedure efficiency, and the scale of production.^30,33^

CNMs have been shown to significantly enhance the properties of magnesium oxychloride cement (MOC). These enhancements in mechanical strength, water resistance, and overall durability make MOC-based CNM-doped composites a promising, ecofriendly, high-performance alternative to traditional Portland cement. The integration of CNMs into MOC not only addresses their inherent limitations, such as low water resistance, but also leverages the unique properties of these nanomaterials to create high-performance construction materials.^34,35^ The addition of multiwalled carbon nanotubes (MWCNTs) and graphene to MOC has been shown to significantly increase both flexural and compressive strength. For instance, the incorporation of these nanomaterials resulted in increases of up to 42.1% in flexural strength and up to 18.2% in compressive strength.^36^ Graphene specifically has been noted to enhance the mechanical resistance and hardness of MOC, making it suitable for load-bearing applications in the construction industry.^37^ One of the primary challenges with MOC is its low water resistance. The inclusion of CNMs, such as MWCNTs and graphene, has been shown to improve water resistance by reducing the average pore diameter and decreasing water absorption by up to 48.2%.^38^ CNMs contribute to a denser microstructure in MOC composites, which is crucial for enhancing both the mechanical and hygric properties. This densification is attributed to the solidification and thickening effects of the nanoadditives, which reduce porosity and improve the overall compactness of the material.^39^

While the integration of CNMs into MOC offers numerous advantages, such as enhanced mechanical properties and durability, it is crucial to consider the cost and scalability of these materials for widespread application. Additionally, determining the optimal CNM content and understanding its influence on both the macroscopic properties and the microstructure of the composites are essential. For these reasons, we prepared a series of MOC samples doped with graphene in concentrations ranging from 0.05 to 1.0 wt %. In our study, we focused not only on evaluating the absolute values of their mechanical parameters but also on examining the associated microstructural phenomena and the mechanisms governing their behavior under mechanical loading.

Despite numerous studies reporting strength enhancement in graphene-modified cementitious materials,^37,38^ the mechanisms governing performance deterioration at higher graphene dosages remain insufficiently understood. In particular, the formation of graphene agglomerates and their influence on pore structure evolution, defect distribution, and fracture behavior are rarely quantified in a systematic manner. Most existing studies focus primarily on peak mechanical properties, while the microstructural thresholds at which graphene addition transitions from reinforcement to structural heterogeneity are not clearly identified, especially in magnesium oxychloride cement systems.

To address these aspects, the present study provides a multiscale investigation explicitly linking graphene dosage, agglomeration morphology, pore structure development, and macroscopic mechanical and hygroscopic performance in MOC composites. The novelty lies in the combined use of conventional microstructural techniques with high-resolution XCT and crack mouth opening displacement (CMOD)-controlled fracture testing, enabling quantitative differentiation between graphene agglomerates and entrapped air voids.

## 2 Experimental Section

### 2.1 Analytical Methods

The phase composition of the prepared samples was studied by using powder X-ray diffraction (XRD). The microstructure of the fracture surface of the prepared samples was studied by using optical microscopy (OM) and scanning electron microscopy (SEM). The fracture surface was also analyzed for its chemical composition using energy-dispersive spectroscopy (EDS). Detailed information about the measurements and equipment is presented in our previous studies.^37,40^

For the hardened samples, structural, microstructural, mechanical, and hygric properties were systematically investigated. The fundamental structural parameters tests included bulk density, specific density, and total open porosity assessment. To characterize the microstructure of graphene-doped MOC composites in greater detail, mercury intrusion porosimetry (MIP) (Pascal 140 and Pascal 440, Thermo Fisher Scientific) was employed. Mechanical performance was assessed in accordance with EN 1015-18,^41^ which provided values of compressive and flexural strength. The dynamic Young’s modulus was determined using an ultrasonic pulse velocity apparatus. Water transport behavior and resistance to water-induced degradation were evaluated by measuring water absorption, residual compressive strength, and the softening coefficient. Further details regarding the applied test procedures and analytical methods are available in refs 38 and 39 .

The fracture tests were performed using a servo-hydraulic testing rig, Instron 8872, with a total capacity of 25 kN. The experimental testing was conducted in a crack mouth opening displacement (CMOD) controlled mode with a loading rate of 0.0025 mm/min. The deformation data were acquired by a high-precision extensometer, model Sandner EXR10-1O, with a measuring range of 1 mm and an accuracy of ±0.1%.

The obtained data served in the evaluation of the work of fracture *W*_f_ and fracture energy *G*_f_. The work of fracture is calculated as the area under the measured Load–CMOD curve, whereas fracture energy is a metric expressing the amount of energy needed to create a new crack surface. The calculation of fracture energy goes by the following equation

$$G_{f}=\frac{W_{f}}{A_{lig}}$$

where *A*_lig_ is the ligament area of the sample.^42–44^

For a detailed microstructural study, X-ray computed microtomography (XCT) analysis was performed to allow full-field inspection of the porous space within the specimen. Radiographical imaging was carried out with a custom-designed XCT setup incorporating a MetalJet D2+ liquid metal anode microfocus X-ray source, a flat-panel detector XRD 3025 with a CsI scintillator, and a high-precision rotary stage equipped with an X-ray-transparent sample mount. The imaging procedure was controlled using in-house-developed real-time software,^45^ ensuring precise synchronization during acquisition of radiographical projections. The selected scanning protocol comprised acquisition of 2800 equiangular projections during full rotation of the sample at a native detector resolution of 3008 × 2512 pixels. The X-ray beam, filtered using a 3 mm layer of aluminum, was generated using an acceleration voltage of 160 kV and 250 W target power. An exposure time of 350 ms was used to capture every radiographic projection, leading to a total duration of the XCT acquisition of approximately 85 min. The cone-beam reconstruction procedure was based on a modified Feldkamp–Davis–Kress cone-beam filtered back-projection algorithm,^46^ implemented in VG Studio MAX 2025.1, resulting in reconstructed 3D images with dimensions of 2512 × 2512 × 3008 and a voxel size of 23 μm.

The reconstructed 3D images were processed to determine the porosity characteristics of the specimens using analytical tools integrated in the reconstruction software. A volume of interest 9VOI) was first generated to eliminate the influence of pores open to the space around the specimen, focusing only on enclosed porosity. Segmentation was carried out using the Otsu thresholding technique^47^ to establish the grayscale boundary between the solid material and air. To isolate and quantify the porous space, a seed-based labeling algorithm was used for pore identification and characterization. This step was followed by morphological filtering to eliminate artifactual or irrelevant features.^48,49^ Only pores encompassing a minimum of 27 voxels were retained to ensure statistical relevance of the results. The porosity evaluation comprised global porosity quantification, planar profiling, distribution mapping, and identification of distinct features, such as graphene clusters and entrapped air voids. The XCT scanning parameters were selected as a compromise between spatial resolution and representative specimen volume. The voxel size of 23 μm allows reliable detection of macroscopic defects and graphene agglomerates exceeding approximately 50–70 μm, while smaller-scale porosity was characterized by MIP. The use of 160 kV acceleration voltage and aluminum filtration minimized beam hardening effects and ensured sufficient penetration through the relatively dense MOC matrix. Segmentation was performed using the Otsu thresholding method to ensure consistent grayscale separation across all specimens. To suppress reconstruction artifacts, only connected features exceeding 27 voxels were retained, corresponding to the smallest statistically reliable detectable entity at the applied voxel resolution. Graphene agglomerates and entrapped air voids were distinguished based on combined volume and morphology criteria (≤0.1 mm^3^ irregular clusters vs ≥0.2 mm^3^ near-spherical voids). Although absolute porosity values may slightly depend on threshold selection, identical criteria applied to all data sets ensure robustness of comparative trends. Since the applied XCT configuration does not provide direct chemical contrast between graphene agglomerates and air-filled voids, the proposed classification should be interpreted as morphology-based rather than as explicit phase identification. Nevertheless, the interpretation was supported by consistent correlations with OM and SEM–EDS observations of graphene-rich clusters.

### 2.2 Composite Sample Preparation

For the preparation of the graphene-doped MOC-based composites, the following chemicals were used: magnesium oxide (**MgO**, purity >98%, Penta, Czech Republic), magnesium chloride hexahydrate (**MgCl**_**2**_ **·6H**_**2**_ **O**, purity >98%, Lachner, s.r.o., Czech Republic), tap water, silica sand in three size fractions 0.0–0.5 mm, 0.5–1.0 mm, and 1.0–2.0 mm (**PG1**, **PG2**, and **PG3**, respectively, Filtrační písky spol. s.r.o., Czech Republic), and graphene nanoplatelets (**G**, specific surface 750 m^2^·g^–1^, Sigma-Aldrich, USA). In all of the samples, the three PG size fractions were used in the weight ratio of 1:1:1. The reference sample with no other additive was termed **REF**; samples containing graphene were termed **G 0.05**, **G 0.1**, **G 0.5**, and **G 1.0** as they contained G in the additional amounts of 0.05, 0.1, 0.5, and 1.0 wt % relative to the amount of MOC, respectively. The selected graphene dosage range (0.05–1.0 wt %) was designed to systematically cover both the low-dosage reinforcement regime commonly reported in the literature (<0.1 wt %) and the higher concentrations where dispersion instability and agglomeration are expected. This range enabled the identification of a potential transition from strength enhancement to agglomeration-driven performance deterioration. The sample mixture compositions are summarized in Table 1. The MgO–MgCl_2_–H_2_O molar ratio was selected to correspond to the stoichiometric composition required for preferential formation of MOC phase 5 (Mg_3_(OH)_5_Cl·4H_2_O), as originally described by Sorel.^50^ Phase 5 is generally considered the mechanically dominant binding phase in MOC systems; therefore, the mixture design was intentionally formulated to promote its formation under ambient curing conditions.

**Table 1:** Sample Mixture Compositions (in g)

	MgO	MgCl_2_·6H_2_O	Water	PG1	PG2	PG3	G
REF	709.90	716.17	444.24	709.90	709.90	709.90	0
G 0.05	709.90	716.17	444.24	709.90	709.90	709.90	0.935
G 0.1	709.90	716.17	444.24	709.90	709.90	709.90	1.870
G 0.5	709.90	716.17	444.24	709.90	709.90	709.90	9.352
G 1.0	709.90	716.17	444.24	709.90	709.90	709.90	18.703

The sample mixing took place in a planetary-type mortar mixer. The first step was the preparation of the MgCl_2_ solution. For the REF sample, the solution was poured into the mortar mixer. For the graphene-doped samples, this solution was used to prepare a graphene suspension, which was homogenized using a rotor–stator mixer at 12,000 rpm for 5 min. This suspension was then poured into the mortar mixer. As the next step, MgO was inmixed at a low speed (140 rpm). Finally, the three PG fractions were added, and the mixture was initially homogenized at low speed and then at high speed (285 rpm). The prepared mortars were poured into prismatic molds with dimensions of 160 mm × 40 mm × 40 mm and left to cure for 1 day. After that, the samples were taken out of the molds and left to cure for an additional 27 days in laboratory conditions (*T* = 23 ± 2 °C, RH = 50 ± 5%).

A schematic of the experimental workflow is shown in Figure 1.

**Figure 1:**
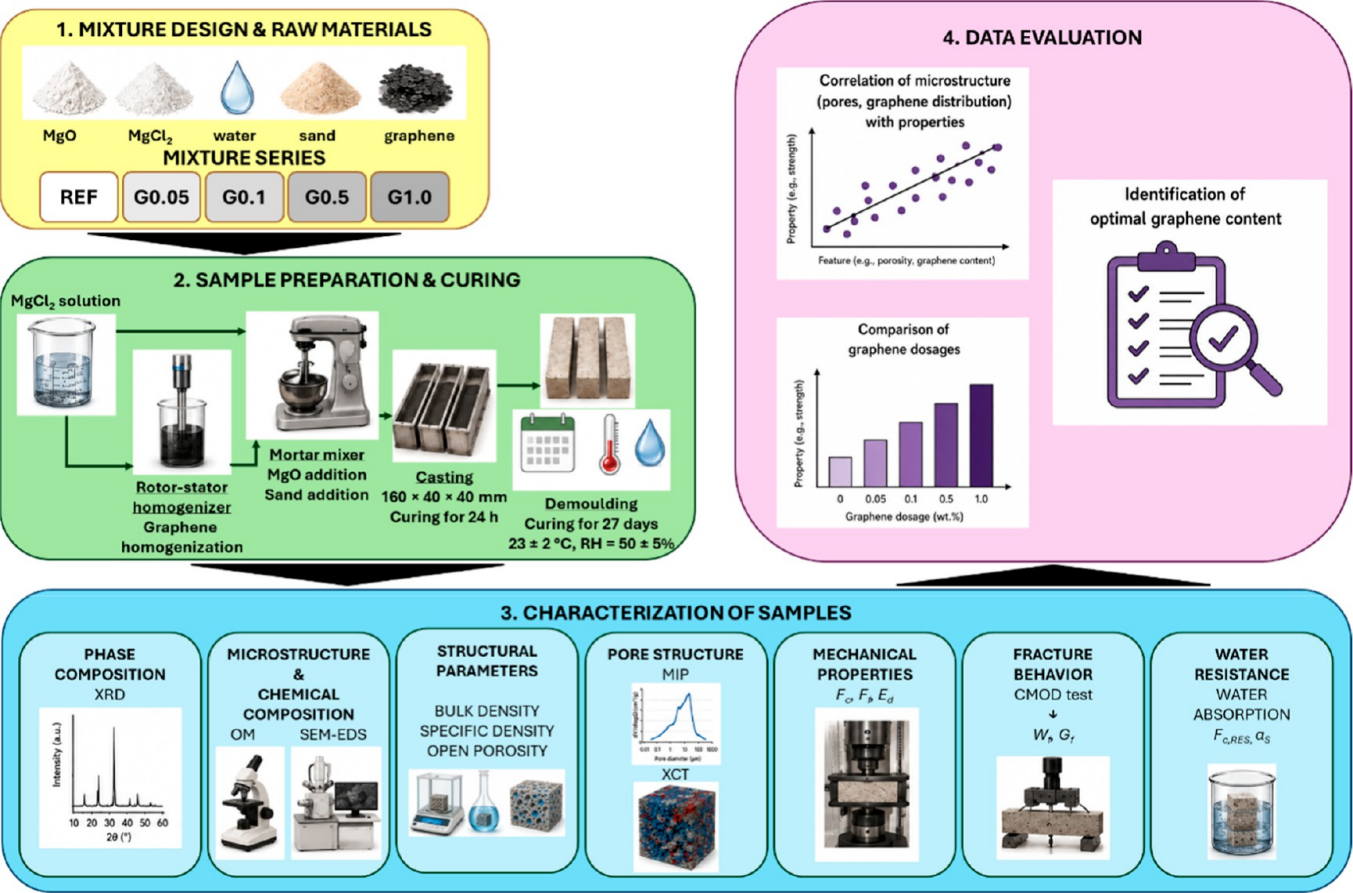
Scheme of the experimental workflow.

## 3 Results and Discussion

Overall, five sets of samples were prepared (Figure 2). The samples became progressively darker as the graphene content increased.

**Figure 2:**
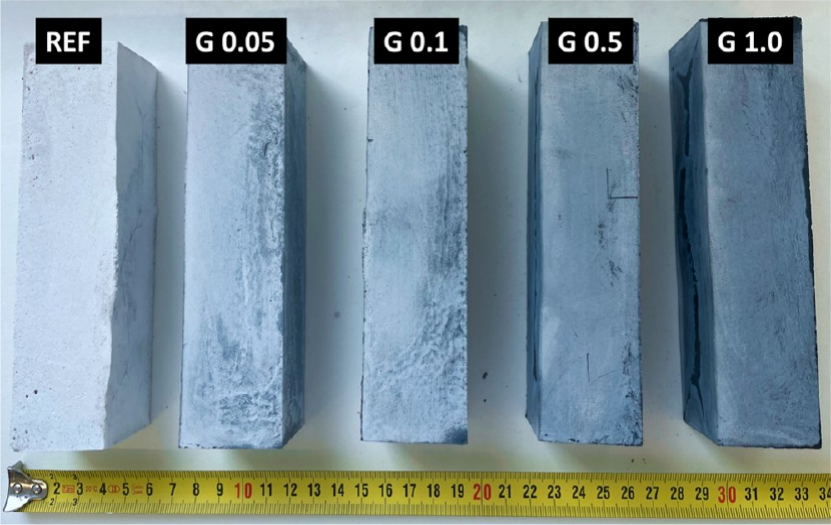
Photograph of the graphene-doped MOC-based composite samples.

### 3.1 Phase Composition and Microstructure

The phase composition of the prepared graphene-doped MOC-based samples was studied by using XRD. The obtained diffraction patterns are listed in Figure 3. All samples show the presence of quartz (ICDD 04-012-0490), originating from the PG filler, and the binder, MOC phase 5 (ICDD 04-014-8836). The graphene nanoadditive cannot be distinguished in the diffraction patterns because its content is low relative to the crystalline phases present.

**Figure 3:**
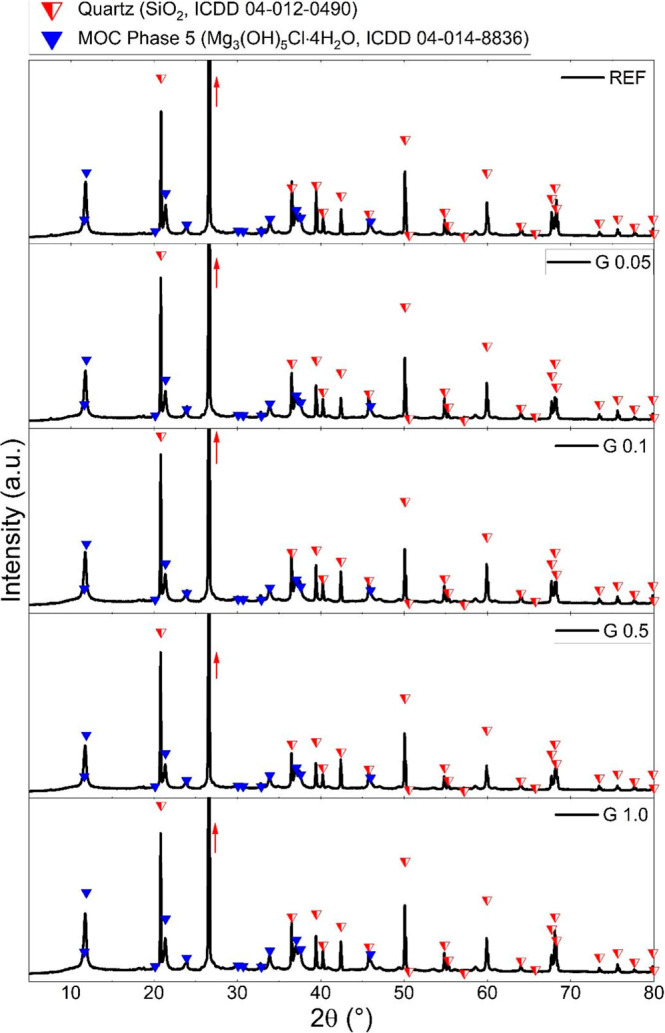
Diffraction patterns of the graphene-doped MOC-based composite samples.

The microstructure of the fracture surface of the prepared samples was studied by SEM. The obtained micrographs (see Figure 4) confirm the presence of the needle-shaped crystals of MOC phase 5. These are very well visible, especially at higher magnification, showing dimensions of 2–10 μm in length and approximately 0.1–0.5 μm in width. These crystals exhibit significant intergrowth and interlocking, the phenomena responsible for the generally good mechanical performance of MOC-based materials.

**Figure 4:**
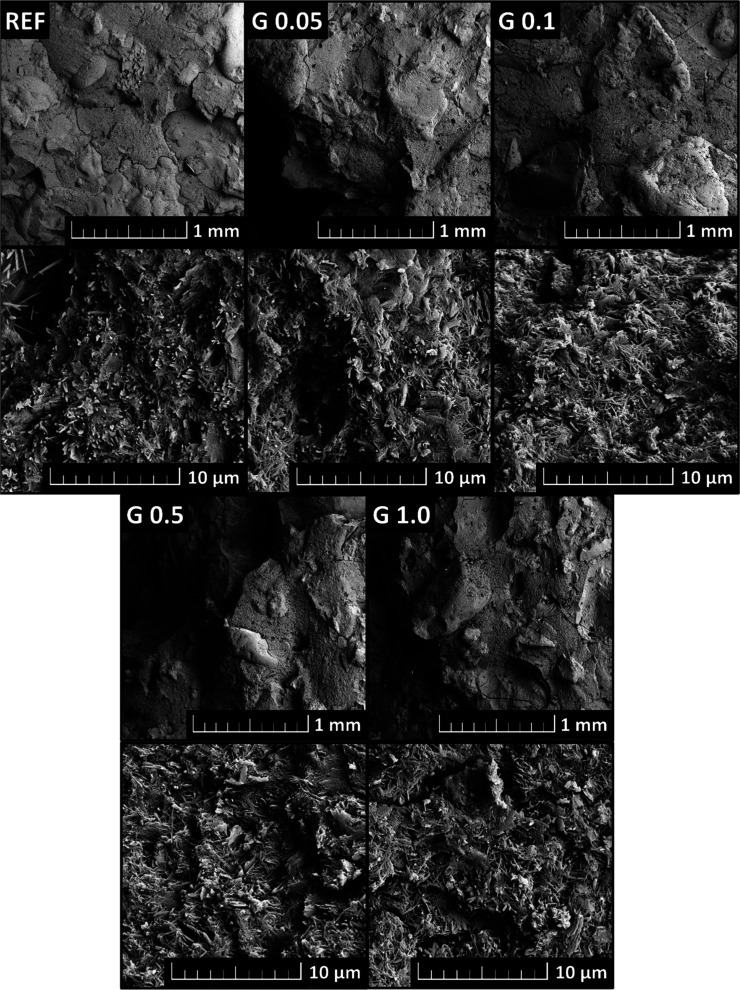
Microstructure of the fracture surface of the MOC–G composite samples.

The microstructure of the samples was further examined to fully assess the graphene homogenization efficiency. The OM micrographs (see Figure 5a) show an increasing graphene agglomerate formation (identifiable as darker irregular clusters) with increasing graphene content. Especially at higher magnification, it is clearly visible that with a higher content of graphene, the number and size of the agglomerates increase. In samples G 0.5 and G 1.0, the size of the agglomerates reaches over 300 μm. The agglomerates were further examined using a SEM–EDS combination. The micrographs (see Figure 5b), together with C elemental maps, confirm the increasing number and size of graphene bundles (bright red C-rich areas in the maps) in the microstructure of the studied composites with increasing G content.

**Figure 5:**
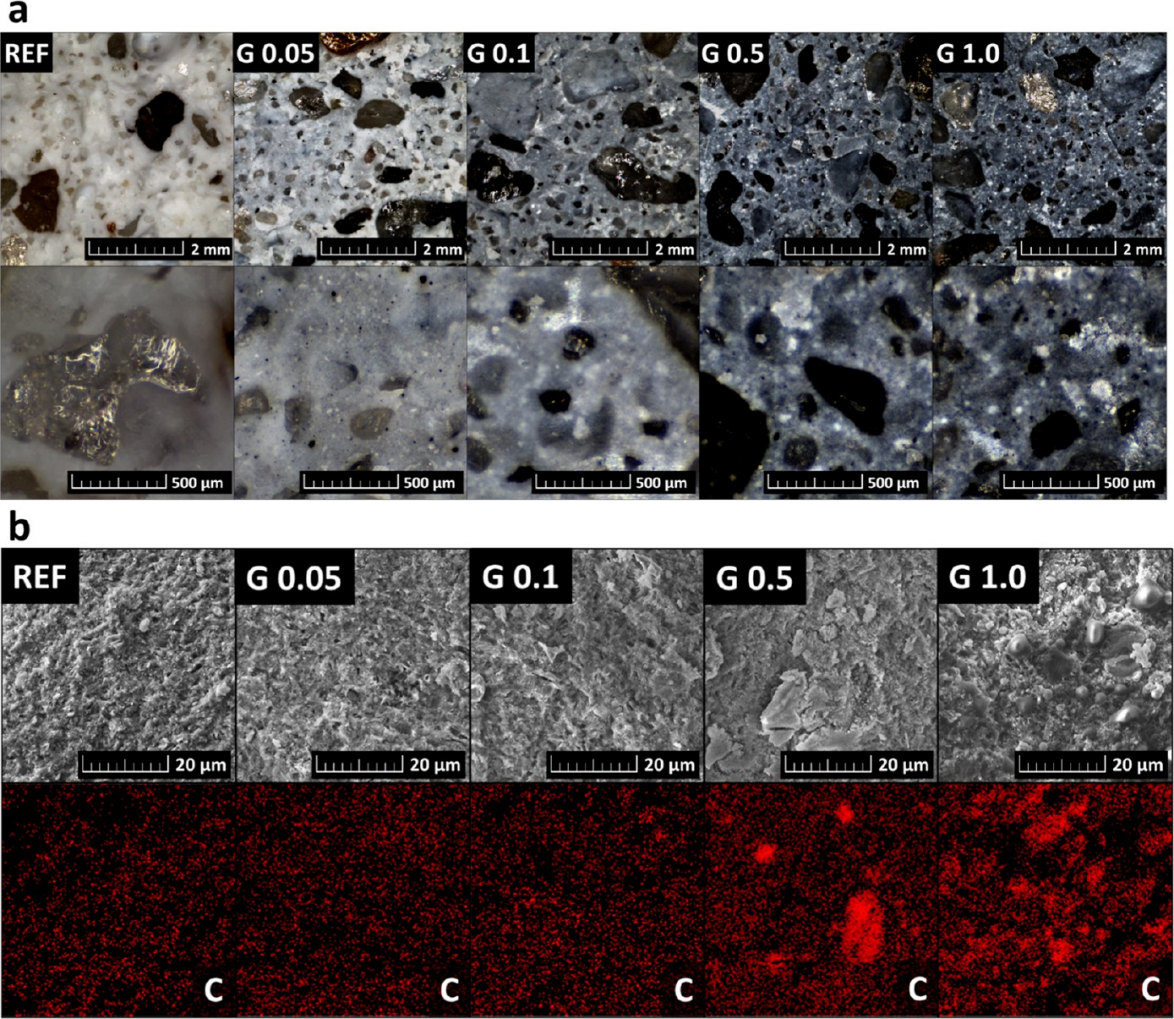
Microstructure of the fracture surface of the graphene-doped MOC-based composite samples obtained from (a) OM and (b) SEM–EDS.

### 3.2 Basic Structural and Hygric Properties

The macrostructural characteristics of the investigated composites are summarized in Table 2. The incorporation of graphene led to an increase in the total open porosity, which can be attributed to the formation of agglomerates of graphene particles, as observed in OM and SEM–EDS analyses. Although graphene was initially well-dispersed in the MgCl_2_ aqueous suspension, its distribution in the MOC mortar was not completely homogeneous. The aggregation of graphene hindered uniform dispersion within the composite matrix, ultimately resulting in a higher porosity. Compared with the sample REF, the increase in porosity ranged from 19.4% to 23.7%. In contrast, the differences in porosity among the graphene-modified composites were minimal, similar to the trends observed for the bulk density and specific density. Owing to the high skeletal density of graphene nanoplatelets,^51,52^ the specific density of the graphene-enriched samples was slightly higher than that of the reference sample.

**Table 2:** Fundamental Structural Parameters of Graphene-Doped MOC-Based Composite Samples, Including Their Expanded Combined Uncertainty

	Bulk density (kg·m^–3^)	Specific density (kg·m^–3^)	Total open porosity (%)
REF	2022 ± 28	2218 ± 31	9.3 ± 0.2
G 0.05	2022 ± 28	2276 ± 32	11.2 ± 0.2
G 0.1	2013 ± 28	2265 ± 32	11.1 ± 0.2
G 0.5	2017 ± 28	2279 ± 32	11.5 ± 0.2
G 1.0	2004 ± 28	2265 ± 32	11.5 ± 0.2

Cumulative and incremental pore size distribution curves obtained by MIP are shown in Figure 6a,b. The MIP data were further used to calculate the total pore volume and average pore diameter, as summarized in Table 3. The pore size distribution curves clearly indicate an increase in porosity for the graphene-modified samples compared with the well-densified reference material REF. The coarsening of the porous structure due to graphene incorporation is also reflected in the total pore volume and the average pore diameter data. Typically, the lowest values of these microstructural parameters were observed for the reference material, while increasing the dosage of graphene nanoplatelets led to higher values. Although the differences among the individual graphene-enriched composites were relatively small, they remained consistently detectable.

**Figure 6:**
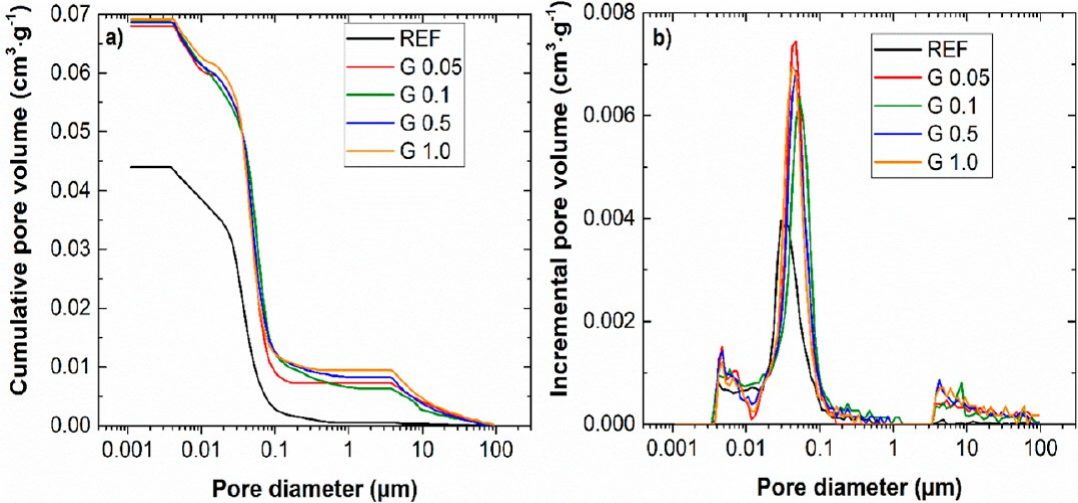
Pore size distribution: (a) cumulative curves; (b) incremental curves.

**Table 3:** Microstructural Parameters of the Graphene-Doped MOC-Based Composites Determined by MIP

	Total pore volume (cm^3^·g^–1^)	Average pore diameter (μm)
REF	0.0441	0.0200
G 0.05	0.0670	0.0257
G 0.1	0.0680	0.0258
G 0.5	0.0688	0.0259
G 1.0	0.0698	0.0276

The increase in the total pore volume detected by MIP indicates that graphene incorporation alters the packing density of the MOC matrix rather than simply filling existing pores. The formation of agglomerates likely creates interfacial transition zones between the graphene clusters and the surrounding matrix, which locally increases the pore volume detected by mercury intrusion. At the same time, the relatively small differences between the individual graphene-modified composites suggest that once agglomeration occurs, the resulting pore structure becomes governed mainly by the cluster morphology rather than by the absolute graphene content. This observation supports the hypothesis that the dispersion state of graphene plays a more critical role than the nominal dose itself.

To further analyze the microstructure of the hardened samples, the individual pore volume fractions are plotted in Figure 6. Water is considered a major disruptive factor for MOC-based materials. In this context, not all pores contribute equally to water transport and related durability risks. Therefore, the concept of effective porosity was adopted, following Mehta and Monteiro, who defined pores larger than approximately 70 nm as active in liquid water transport.^53,54^ Conversely, pores larger than approximately 10 μm are dominated by gravitational forces rather than capillary rise. Accordingly, the effective porosity in the present study was defined as the volume fraction of pores within the 0.1–10 μm range, as determined by MIP analysis. This pore size range is highlighted in red in Figure 7. This parameter was introduced to better quantify the fraction of pore space directly involved in capillary water ingress and potential deterioration mechanisms. An increase in effective porosity indicates a higher capacity for capillary-driven water transport, which may accelerate moisture-related degradation processes.

**Figure 7:**
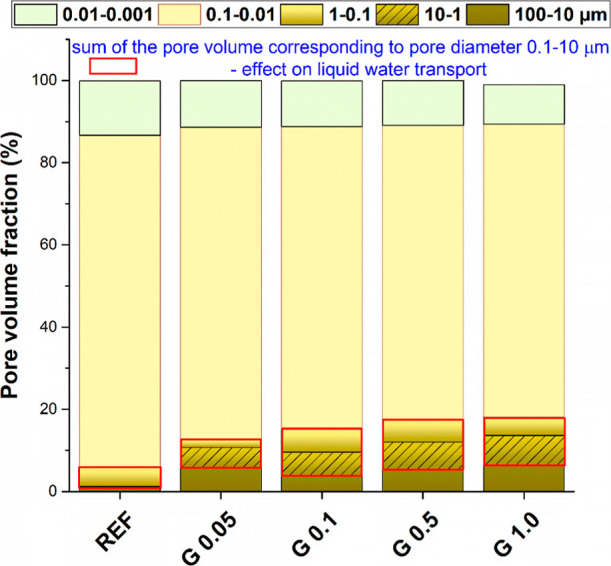
Pore volume fraction determined by MIP.

The calculated effective porosity for water transport was the lowest in the reference composite (5.1%), whereas it increased in graphene-containing materials, consistent with the pore size distribution curves and other microstructural observations. Specifically, the effective porosities were 6.94%, 11.3%, 12.4%, and 11.8% for samples G 0.05, G 0.1, G 0.5, and G 1.0, respectively. These results are consistent with the water absorption data for graphene-modified composites, as shown in Table 4. However, despite the higher effective porosity, water absorption was reduced in the graphene-containing samples due to the hydrophobic nature of graphene nanoparticles,^55^ which outweighed the effect of increased pore volume on water ingress.

**Table 4:** Water Absorption of the Graphene-Doped MOC-Based Composites

	24 h water absorption by mass (%)	24 h water absorption by volume (kg·m^–3^)
REF	3.01 ± 0.04	55.02 ± 0.77
G 0.05	2.75 ± 0.04	53.31 ± 0.75
G 0.1	2.82 ± 0.04	53.74 ± 0.75
G 0.5	2.88 ± 0.04	57.53 ± 0.81
G 1.0	2.91 ± 0.04	55.38 ± 0.78

Although the effective porosity increased with higher graphene dosages, the reduction in 24 h water absorption suggests that pore connectivity and surface chemistry play a dominant role in water transport behavior. The hydrophobic nature of graphene nanoplatelets likely reduces the wettability of internal pore surfaces, thereby limiting capillary water ingress despite the presence of transport-active pores. In addition, agglomerates may locally disrupt pore continuity, leading to a partial interruption of connected capillary pathways.

Compared to conventional approaches for improving MOC water resistance—such as silane treatment or incorporation of pozzolanic materials^56,57^ —graphene does not primarily densify the matrix but instead modifies surface interactions and transport mechanisms at the microstructural level. While polymer additives typically reduce porosity and increase matrix compactness, graphene appears to influence water transport predominantly through surface energy effects and altered pore connectivity.

### 3.3 Mechanical Performance

The mechanical parameters of composites cured for 28 days are presented in Figure 8 and Table 5. At the lowest graphene nanoplatelet concentration, an increase in compressive strength was observed relative to the reference material. However, at higher graphene dosages, both compressive and flexural strengths decreased, which corresponded with the increased porosity and reduced bulk density. This effect was attributed to the agglomeration of graphene at higher content, which impaired the homogeneous dispersion of reinforcing elements within the composite matrix. Our results are consistent with the reduction in mechanical strength reported by Chetty et al.,^58^ who investigated the influence of graphene dosage on the mechanical performance of concrete. The authors identified the optimal nanoadditive content in the range of 0.02–0.07%. These findings highlight that the dosage of nanoreinforcing particles must be carefully optimized on a case-by-case basis and should not exceed the percolation threshold. The lowered Young’s modulus further indicates a decrease in stiffness of the graphene-modified materials, rendering them less brittle and more flexible under mechanical loading.

**Figure 8:**
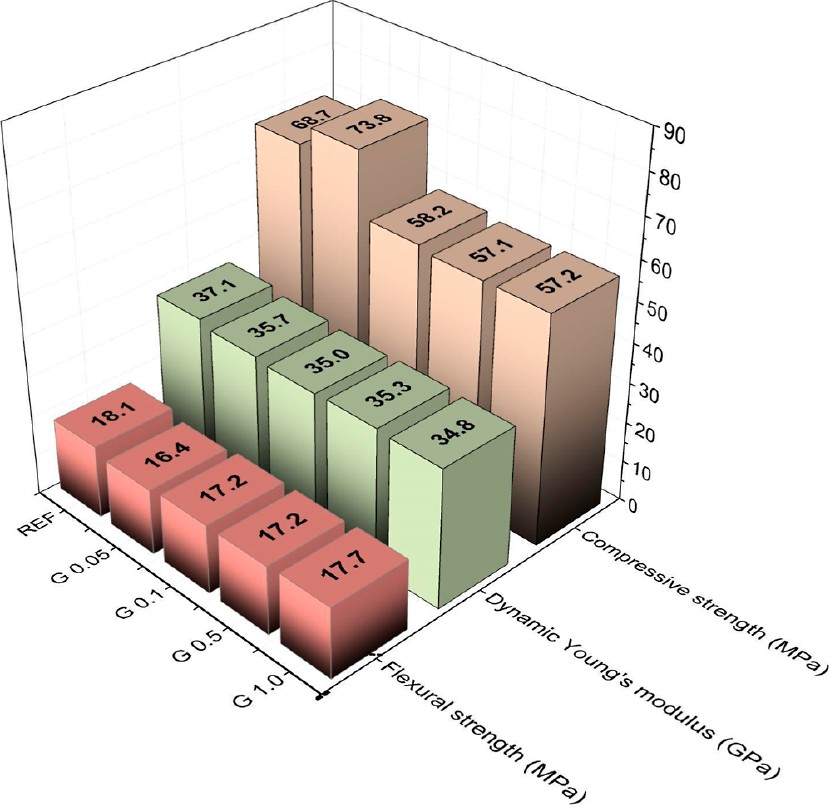
Mechanical parameters of the prepared G-doped MOC composites.

**Table 5:** Mechanical Parameters of G-Doped MOC Composites, Including Expanded Combined Uncertainty Data

	Flexural strength (MPa)	Compressive strength (MPa)	Dynamic Young’s modulus (GPa)
REF	18.1 ± 0.3	68.7 ± 1.0	37.1 ± 0.9
G 0.05	16.4 ± 0.2	73.8 ± 1.0	35.7 ± 0.8
G 0.1	17.2 ± 0.2	58.2 ± 0.8	35.0 ± 0.8
G 0.5	17.2 ± 0.2	57.1 ± 0.8	35.3 ± 0.8
G 1.0	17.7 ± 0.2	57.2 ± 0.8	34.8 ± 0.8

The evolution of mechanical properties reflects the competing effects of nanoreinforcement and agglomeration-induced heterogeneity. At low graphene dosage (0.05 wt %), well-dispersed nanoplatelets can bridge microcracks and improve stress transfer within the MOC matrix, which explains the observed increase in compressive strength. However, with increasing graphene content, the probability of particle–particle interaction rises, leading to the formation of micron-scale agglomerates, as confirmed by OM, SEM–EDS, and XCT observations. These agglomerates act as local heterogeneities and stress concentrators that disrupt the continuity of the MOC matrix and reduce the efficiency of load transfer between the matrix and the nanomaterial. Similar behavior has been reported for graphene- and CNT-modified cementitious materials, where excessive nanomaterial dosage leads to agglomeration and deterioration of mechanical performance due to microstructural defects and increased pore connectivity.^23,58–61^

The resistance of the investigated materials against water-induced damage is summarized in Table 6. Partial suppression of water ingress enhanced the residual strength of the graphene-modified samples relative to the reference material. Likewise, the softening coefficient, reflecting the preservation of mechanical performance—specifically compressive strength—was improved with increasing graphene dosage in the composites. This improvement is attributed to graphene nanoplatelets that hinder crack propagation by impeding the disintegration and dilution of MOC phase 5. At the same time, the hydrophobic nature of graphene reduces the wettability of internal pore surfaces and limits capillary water ingress, which contributes to the improved durability response of the graphene-modified composites. Although agglomeration of graphene at higher dosages introduces microstructural heterogeneities, these effects appear to be partially compensated by the hydrophobic surface interactions of graphene within the pore system, resulting in improved residual strength after water exposure.

**Table 6:** Residual Compressive Strength and Softening Coefficient of the Graphene-Doped MOC-Based Composites and Their Expanded Combined Uncertainties

	Residual compressive strength (MPa)	Softening coefficient (%)
REF	44.9 ± 0.6	65.4 ± 1.3
G 0.05	49.5 ± 0.7	67.1 ± 1.3
G 0.1	45.3 ± 0.6	77.9 ± 1.6
G 0.5	51.0 ± 0.7	89.4 ± 1.8
G 1.0	48.1 ± 0.7	85.6 ± 1.7

### 3.4 Fracture Behavior

Figure 9 displays the recorded load–CMOD response obtained from a static fracture test using a three-point bending geometry with an initial notch at the midspan of the sample. The notch had a depth of approximately 12 mm, which is 1/3rd of the sample’s height. The measured fracture responses exhibit a high degree of consistency and homogeneity across all investigated materials. This is more pronounced in the initial linear–elastic phase of measured load–CMOD curves, as well as in the postpeak softening branch. The recorded maximum load is approximately 1.43 kN for all measured samples and produces a CMOD of approximately 0.02 mm. The measured postpeak decreasing branch of the load-bearing capacity is typical for quasi-brittle materials.

**Figure 9:**
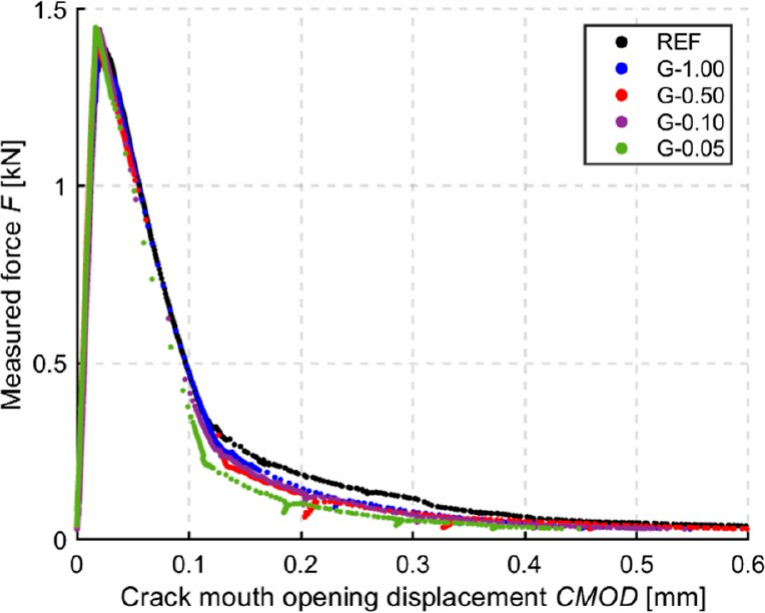
Load–CMOD curves for studied materials.

Using the experimental data in Figure 8, one can calculate the work of fracture, *W*_f_, and the fracture energy, *G*_f_. These metrics help quantify the fracture process and enable a comparison across materials. The evaluated work of fracture *W*_f_ is shown in Figure 10a, while the comparison of the evaluated fracture energy *G*_f_ is shown in Figure 10b.

**Figure 10:**
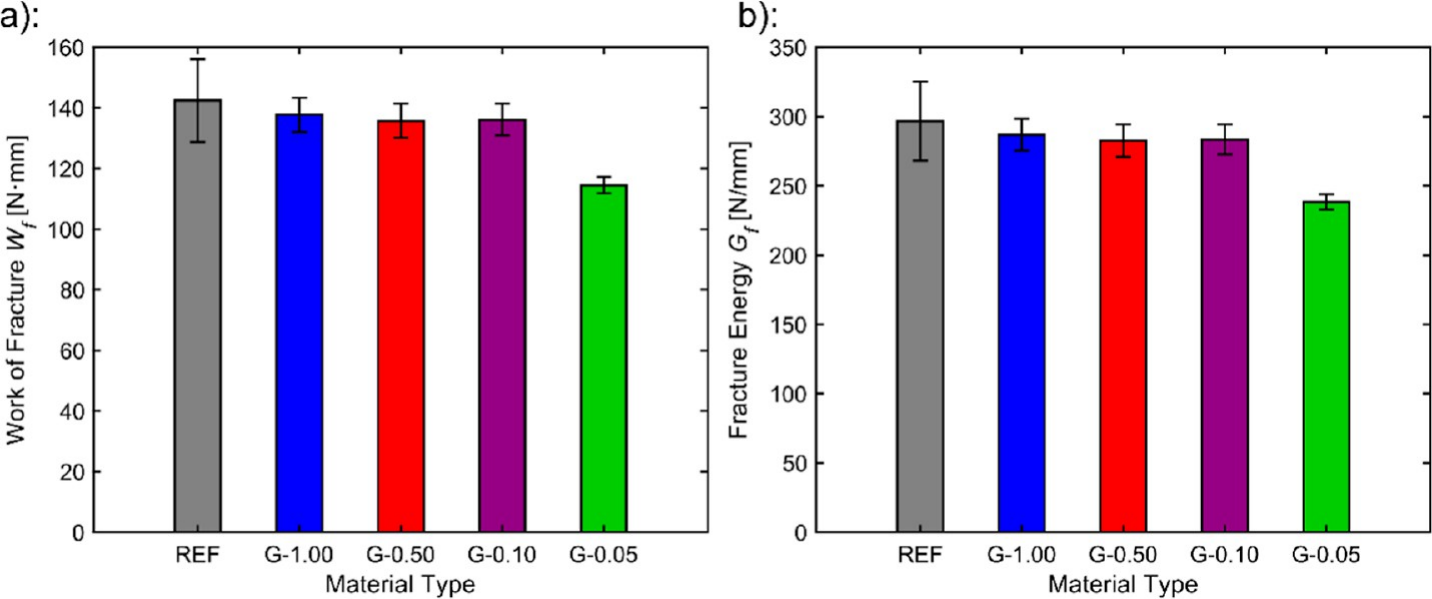
Results of fracture test: (a) work of fracture *W*_f_ and (b) fracture energy *G*_f_.

The obtained values of *W*_f_ and *G*_f_ show similar homogeneous behavior across all studied materials, with average values of 148 N·mm and 290 N·mm^–1^, respectively. The samples with low graphene content, such as G 0.05, showed the lowest values of measured fracture properties. A measured decrease in fracture energy *G*_f_ is likely associated with partial agglomeration of graphene particles during mixing, as presented in Figure 4 (OM and EDS of fracture surfaces), which locally disrupts matrix densification and results in additional pore formation.

### 3.5 XCT Analysis

To obtain insight into the internal structure of the investigated specimens, a detailed microstructural analysis was performed using volumetric data obtained from XCT. This analysis enabled evaluation of the porosity distribution and morphological features of the internal void space across all spatial directions in the samples. The figures below illustrate a 3D visualization of the identified pores within a semitransparent volume to visualize the spatial arrangement of internal features, together with the corresponding sphericity–equivalent diameter plot. Figure 11 presents the results for the REF specimen, with those for the G-containing specimens shown in Figure 12a–d.

**Figure 11:**
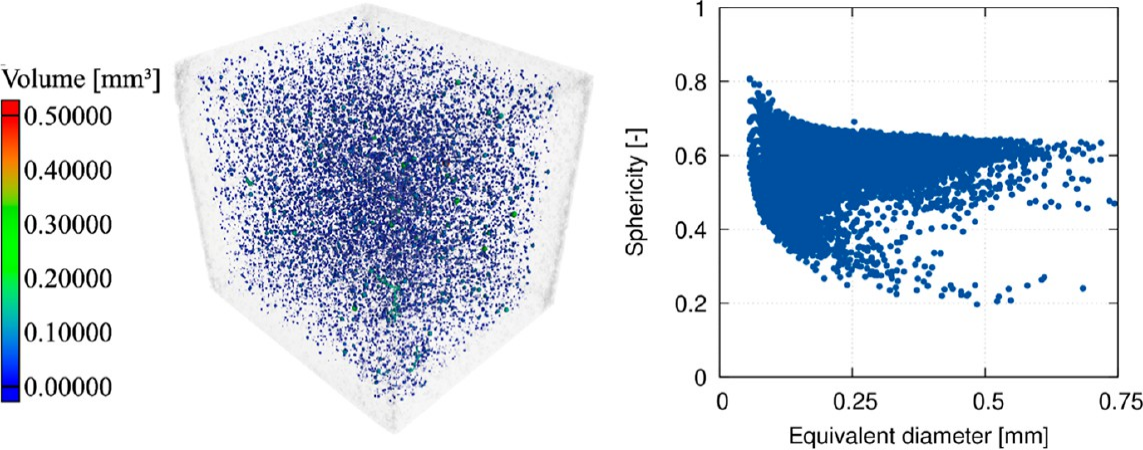
3D visualization of the identified pores within the microstructure of specimen REF and the corresponding sphericity–equivalent diameter plot.

**Figure 12:**
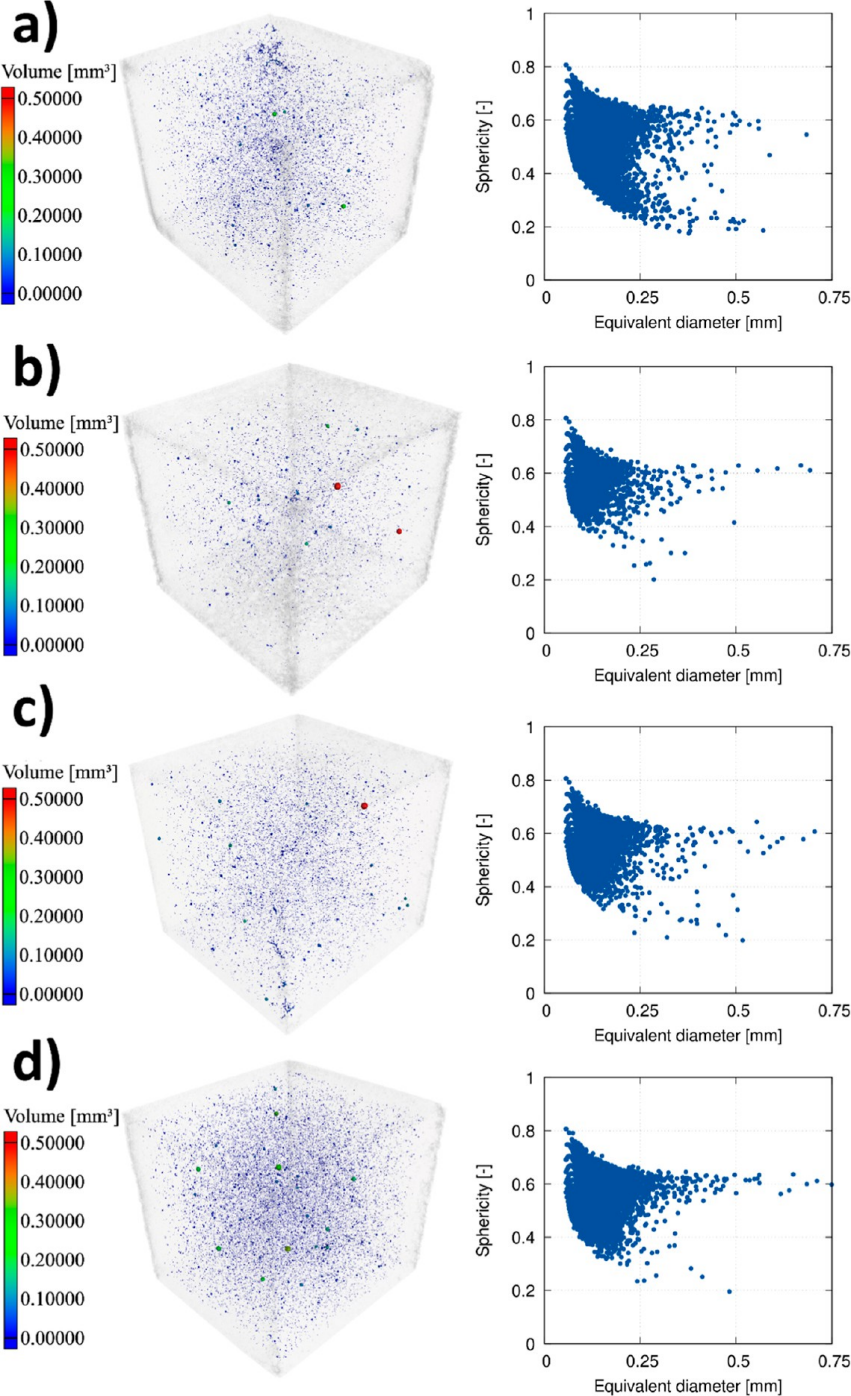
3D visualization of the identified pores in the microstructure of specimens (a) G 0.05, (b) G 0.1, (c) G 0.5, and (d) G 1.0 and the corresponding sphericity–equivalent diameter plots.

Based on volumetric segmentation and morphological classification, individual entities with volumes up to 0.1 mm^3^ and highly irregular shapes (colored blue) are interpreted as graphene agglomerates in samples shown in Figure 12a–d. In contrast, spherical or near-spherical features with volumes exceeding 0.2 mm^3^ (colored green, orange, and red) are in these cases considered entrapped air voids. These two types of porosity differ markedly in size, shape, and likely origin, providing valuable insights into graphene dispersion and air entrapment during specimen preparation. Although the applied XCT approach does not allow direct chemical identification of individual features, the observed differences in morphology, sphericity, and spatial distribution were consistent across all investigated specimens and correlated well with OM and SEM–EDS observations. The proposed classification should therefore be understood as a morphology-based interpretation of the detected heterogeneities rather than as an unambiguous phase determination.

The reconstructed XCT data sets allow quantitative assessment of void morphology and its evolution with increasing graphene weight fraction. The reference sample (REF) exhibits predominantly spherical or near-spherical voids with the sphericity dominantly in the range of 0.5–0.8 for pores with equivalent diameter up to the largest pores, as documented by higher sphericity values in the sphericity–equivalent diameter plots (Figure 10). These defects are characteristic of entrapped air formed during mixing and casting stages of material preparation.

In contrast, specimens containing graphene (Figure 11a–d) show distinctively different morphological characteristics of the void space, composed of irregular, low-sphericity pores in majority, bounded from above by an equivalent diameter of 0.25 mm, corresponding to the presence of graphene agglomerates. Here, the sphericity of the small pores is in the range of approximately 0.3 to 0.6, while larger pores form clusters around low and high sphericity values of 0.2–0.3 and 0.6, respectively. For the graphene content in the range of 0.1 to 1.0 wt %, the relative contribution of small irregular pores increases proportionally with graphene content. However, the sphericity–equivalent diameter plot of G 0.05 sample shows a distinctive difference, particularly in comparison with the G 0.1 sample, indicating that such a low amount of graphene leads to a microstructural response that is a transition between the reference, graphene free, material, and material with higher graphene content. These findings indicate that above approximately 0.1 wt % of graphene, dispersion-related heterogeneity becomes a governing microstructural factor.

The observed MIP and XCT results exhibit scale-dependent differences, where MIP indicates an increase in total and effective porosity with increasing graphene content and XCT-based directional porosity reveals a reduction in large entrapped voids relative to the reference material. This apparent discrepancy can be explained by the different detection scales of the two methods. XCT captures macroscopic voids and agglomerates above the voxel resolution of approximately 50–70 μm, whereas MIP is sensitive to capillary pores in the submicron to micrometer range. The incorporation of graphene thus appears to reduce large entrapped air voids while simultaneously promoting microstructural heterogeneity at smaller scales, leading to pore coarsening detected by MIP. The incorporation of graphene thus appears to reduce large entrapped air voids while simultaneously promoting microstructural heterogeneity at smaller scales, leading to pore coarsening detected by MIP. Therefore, the combined use of MIP and XCT is necessary to characterize the full pore-size spectrum present in graphene-modified MOC composites, ranging from submicron capillary pores to tens- and hundreds-of-micrometer-scale macrovoids and graphene agglomerates. The complementary nature of both techniques enables a more reliable interpretation of the pore system evolution than either method alone.

The fracture results shown in Figure 9 reflect these microstructural changes. While mixtures with higher graphene contents (G-1.00, G-0.50, and G-0.10) exhibit fracture parameters broadly comparable to those of the reference mixture, the G-0.05 specimen shows a noticeable reduction in both work of fracture and fracture energy. This suggests that the transition microstructure observed at low graphene dosage is less effective in resisting crack initiation and propagation, likely due to increased local heterogeneity and stress concentration sites associated with an irregular pore morphology. In this sense, the fracture response appears to depend not only on total porosity but also on the size, shape, and spatial distribution of graphene-related defects.

This behavior is consistent with observations reported for unmodified MOC and other cementitious composites containing nanomodifiers, where insufficient dispersion may suppress potential toughening effects^62–64^ and instead promote pore formation and microstructural nonuniformity. The present results therefore indicate that the fracture performance of graphene-modified MOC is strongly governed by the quality of graphene dispersion and the resulting defect topology.

The obtained XCT results provide microstructural context for the measured fracture behavior. The relatively homogeneous distribution of voids and agglomerates in samples G 0.1–G 1.0 is consistent with the experimentally observed stability of fracture energy (∼290 N·mm^–1^). The agglomerates are not observed to create continuous planar discontinuities but instead occur as isotropically dispersed volumetric heterogeneities within the matrix, a morphology consistent with the quasi-brittle fracture response. At higher weight fractions (≥0.5 wt %), the increasing size and frequency of irregular pore clusters coincide with the reduction in compressive strength, despite the improved residual performance after water exposure.

Evaluation of directional porosity variability reveals systematic changes in the spatial distribution of macrodefects with increasing graphene content. The reference material exhibits steeper porosity gradients in the vicinity of the outer face of the specimens along the *X*- and *Y*-directions, which may be associated with casting-induced air accumulation. Resulting from the presence of graphene in the mixture, a more uniform directional porosity distribution over a larger interior volume of samples indicates altered rheological behavior during mixing and casting, potentially due to the increased viscosity of the graphene suspension. This observation indicates a reduced macroscopic air entrapment.

In addition to calculating the overall porosity of the sample, the XCT data enable the analysis of unidirectional porosity. Unidirectional porosity provides information about the setting of the material during the manufacturing process and helps identify the locations with higher pore/defect accumulation. The results of unidirectional porosity in the *X*-, *Y*-, and *Z*-directions are presented in Figure 13. A careful examination of Figure 13 reveals a decrease in the porosity with increasing graphene content in all investigated directions. The reference sample REF exhibits the highest pore content, which can be attributed solely to the presence of MOC phase 5. The graphene agglomerates significantly reduce the pore content from approximately 0.6% to below 0.1%. This reduction in porosity is evident when the average directional porosity is calculated.

**Figure 13:**
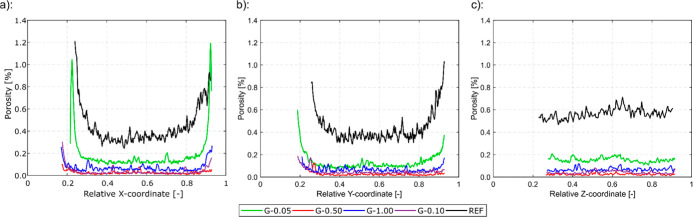
Results of directional porosity along the relative coordinates: (a) *X*-direction, (b) *Y*-direction, and (c) *Z*-direction.

Figure 14 shows the average directional porosities evaluated for each studied material. The reference sample has the highest pore content, which is more pronounced in the *Z* direction, of approximately 0.6%. The G 0.05 sample has the lowest graphene content, as documented by the highest porosity in graphene-enhanced samples, with a porosity of approximately 0.2%. Furthermore, a lower degree of graphene agglomeration yields a low-density microstructure, consistent with the lowest measured *G*_f_ value. Such fracture behavior and pore content are outside the observed relatively homogeneous behavior, from which the optimal graphene content to improve micro- and macroresponse is >0.1%.

**Figure 14:**
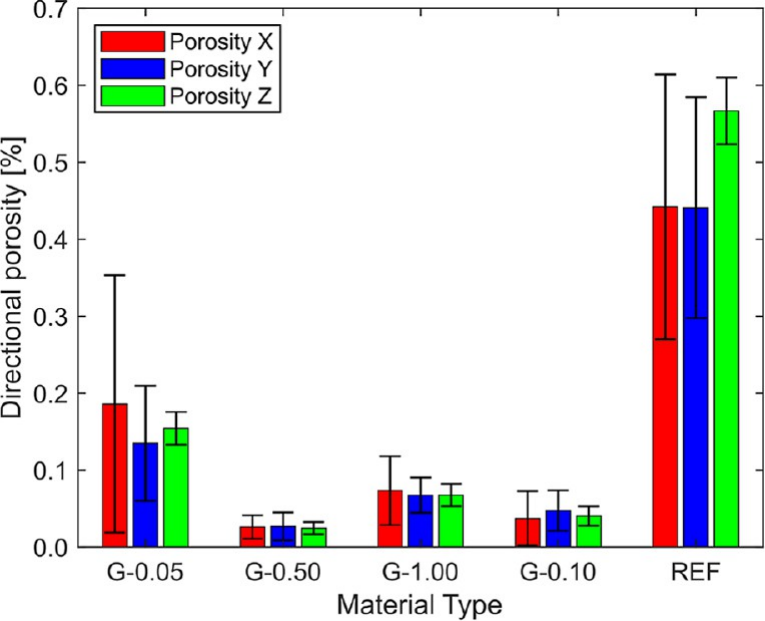
Average directional porosity with standard deviation for studied materials.

It should be noted that the applied XCT resolution does not allow visualization of individual graphene nanoplatelets or submicrometer pore structures. Therefore, the analysis focuses on agglomerates and macrostructural heterogeneities that can be detected within the given voxel size. Furthermore, segmentation using the Otsu thresholding method introduces inherent sensitivity to the grayscale distribution, which may slightly influence absolute porosity values. Nevertheless, comparative trends among specimens remain robust and reproducible.

From a practical perspective, the observed agglomeration behavior indicates that microstructural control in graphene-modified MOC systems is highly sensitive to dispersion quality and mixing conditions. While laboratory-scale homogenization enables partial stabilization of low graphene dosages, scaling to industrial production may introduce variability due to differences in shear energy, mixing time, and air entrainment. In particular, preventing agglomerate formation above the identified dosage threshold requires controlled dispersion protocols that remain reproducible at larger batch volumes.

Furthermore, the economic feasibility of graphene incorporation must consider not only material cost but also processing energy and quality control requirements. Therefore, practical implementation will depend on developing robust dispersion strategies and verifying the performance in larger structural elements under realistic service conditions.

### 3.6 Engineering Implications and Potential Applications

The results obtained in this study provide useful guidance for the engineering application of graphene-modified magnesium oxychloride cement composites. The identified optimal graphene dosage range (≤0.1 wt %) allows enhancement of compressive strength and reduction of water absorption without introducing significant structural heterogeneity. Materials with such a composition may therefore be suitable for use in prefabricated elements, repair mortars, or thin-layer construction systems where improved durability and mechanical performance are required.

The improved resistance to water-induced degradation observed for graphene-containing composites also suggests potential applications in environments exposed to intermittent moisture or moderate humidity fluctuations, such as interior structural components, flooring systems, or restoration materials used in building conservation. At the same time, the present study demonstrates that excessive graphene dosage leads to agglomeration and microstructural instability, which may negatively affect the mechanical performance. From an engineering perspective, careful control of nanomaterial dispersion and dosage is therefore essential for ensuring reliable material performance and reproducibility in practical construction applications.

## 4 Conclusions

This study provides multiscale insight into the role of graphene in magnesium oxychloride cement (MOC) composites, with a particular emphasis on establishing direct links between microstructural evolution and mechanical, hygric, and fracture performance. By combining conventional characterization techniques with high-resolution XCT, we elucidated the mechanisms governing the macroscopic response of graphene-doped MOC.

The following key findings were reached:

- Graphene dosage ≤0.1 wt % preserved mechanical performance and reduced 24 h water absorption from 3.01% to 2.75%.
- Agglomeration at ≥0.5 wt % formed clusters >300 μm and increased total open porosity from 9.3% to 11.5%.
- The effective transport porosity increased from 5.1% to 12.4%, indicating pore structure coarsening.
- The residual compressive strength reached 51.0 MPa, and the softening coefficient improved from 65% to 89%.
- Fracture energy remained stable at ∼290 N·mm^–1^, confirming preservation of quasi-brittle behavior.
- Multiscale XCT analysis enabled quantitative differentiation between graphene agglomerates and entrapped air voids.

Overall, the results demonstrate that the performance of graphene-doped MOC composites is governed by microstructural stability and dispersion quality rather than graphene content alone. This study uniquely combines MIP, XCT, and CMOD-controlled fracture testing to distinguish graphene agglomerates from entrapped air voids and to establish clear structure–property relationships. These findings move beyond empirical optimization and provide a mechanistic framework for the rational design of durable, high-performance, graphene-enhanced MOC composites for sustainable construction applications. From an engineering perspective, the study establishes practical dosage limits for the investigated system. However, scalability remains dependent on reproducible dispersion techniques, control of air entrainment during mixing, and economic feasibility of graphene incorporation at larger production volumes. Future research should focus on dispersion stabilization strategies, surface functionalization approaches, and validation in larger structural elements to assess long-term durability and industrial applicability.

## Data Availability

The data underlying this study are not publicly available due to intellectual property considerations. The data set forms part of an ongoing research and development project on magnesia-based composites modified with nanoadditives, for which patent protection and other application outcomes are currently being pursued. The data are available from the corresponding author upon reasonable request and subject to any applicable confidentiality and intellectual property restrictions.
